# Many-Body Dynamics and Decoherence of the *XXZ* Central Spin Model in External Magnetic Field

**DOI:** 10.3390/e22010023

**Published:** 2019-12-23

**Authors:** Xu Zhou, Qing-Kun Wan, Xiao-Hui Wang

**Affiliations:** 1 School of Physics, Northwest University, Xi’an 710127, China; 2 Institute of Modern Physics, Northwest University, Xi’an 710127, China; 3 Shaanxi Key Laboratory for Theoretical Physics Frontiers, Xi’an 710127, China

**Keywords:** many-body dynamics, solvable models, central spin model, exact diagonalization, fidelity, disordered field

## Abstract

The many-body dynamics of an electron spin−1/2 qubit coupled to a bath of nuclear spins by hyperfine interactions, as described by the central spin model in two kinds of external field, are studied in this paper. In a completely polarized bath, we use the state recurrence method to obtain the exact solution of the XXZ central spin model in a constant magnetic field and numerically analyze the influence of the disorder strength of the magnetic field on fidelity and entanglement entropy. For a constant magnetic field, the fidelity presents non-attenuating oscillations. The anisotropic parameter λ and the magnetic field strength *B* significantly affect the dynamic behaviour of the central spin. Unlike the periodic oscillation in the constant magnetic field, the decoherence dynamics of the central spin act like a damping oscillation in a disordered field, where the central spin undergoes a relaxation process and eventually reaches a stable state. The relaxation time of this process is affected by the disorder strength and the anisotropic parameter, where a larger anisotropic parameter or disorder strength can speed up the relaxation process. Compared with the constant magnetic field, the disordered field can regulate the decoherence over a large range, independent of the anisotropic parameter.

## 1. Introduction

Coherence, as a quantum property from the quantum states superposition principle, which marks the departure of quantum theories from the principles of classical physics, is the origin of the power of quantum information processing. However, in the real world, nearly all quantum systems inevitably interact with the environment, which may cause decoherence. In 2000, DiVincenzo put forward five requirements for the physical realization of quantum computers [1], one of which pointed out that the system requires long coherence times for quantum operations to be completed within. Therefore, maintenance of the quantum coherence property for a long time without damage from the external environment has always been a focus of research in the field of quantum computing and quantum information [2,3]. As one of the possible candidates for a qubit, the electron spin of a semiconductor quantum dot can achieve single-electron readout and coherent control [4,5,6,7], but leads to electron spin decoherence due to the existence of nuclear spins in the substrate and the hyperfine interactions between the electron spin and the nuclear spins [8,9]. Generally, in bulk solid materials, the decoherence of an electron spin is usually based on spin-orbit coupling. However, the relaxation [10] and dephasing [11] caused by spin orbits are strongly suppressed in a quantum dot; thus, the hyperfine interactions become a major obstacle for maintaining long coherence times. The many-body dynamics of single-electron spin coupled to a nuclear spin bath of non-interacting spins is described by the Gaudin’s central spin model (CSM) [12], and the Hamiltonian is

(1) $$H_{\mathrm{CSM}}=\sum_{i=1}^{K}A_{i}S_{0}\cdot S_{i},$$

where *K* is the number of bath spins and $A_{i}$ is the coupling strength. It is a suitable model to describe the dynamics of the electron spin, which is being used widely to model the hyperfine interaction between an electron spin and the surrounding nuclear spins. This type of interaction is inevitable in a solid system which has unpaired electrons, such as some point defects (localized crystal imperfections), most transition-metal ions, and most rare-earth ions [13]. Thus, this model has been applied in the systems of quantum dots, NV centers, and semiconductors. Furthermore, understanding the many-body dynamics of CSM will play a crucial role in regulating quantum states [14,15,16]. Several techniques have been developed to analyze dynamics of this model, including Bethe ansatz [12,17,18,19], exact diagonalization [20,21], Chebyshev expansion (CE) [22,23], Density-matrix renormalization group (DMRG) [24,25], and non-Markovian master equation methods [26,27].

Due to the exponentially growing complexity, quantum many-body dynamics is always a difficult question in physics, but the exact solutions of integrable models break this limitation and allow us to study the dynamics of any size [28,29]. The CSM, as a typical integrable system, has exact solutions which can be derived by the Bethe ansatz method [12,17,18,19]. It should be mentioned that in [30], the author pointed out the relationship between the spontaneous symmetry breaking and the quantum Yang-Baxter equations, which inspired us to understand the many-body dynamics from the symmetry in integrable system. What’s more, many researchers in the literature have paid attention to the CSM with homogeneous couplings in a constant magnetic field. It has been found that the central-spin polarization recurs after the recurrence time $\tau_{p}=\pi \;\left(2\pi \right)$ for an even (odd) particle number [18], and the evolution of the spin polarization has an oscillatory form [19]. Recently, in [31], the author investigated the characteristics of many-body localization (MBL) in the CSM. A series of counter-intuitive phenomena caused by MBL [32] have challenged our understanding of statistical mechanics [33,34]. Moreover, the MBL provides a generic approach to prevent a system from thermalization [35,36,37] and to break its ergodicity [38]. In most cases, a disordered field is a key factor for the phase transition from ETH to MBL [39,40,41]. The above observations have inspired us to discuss the central spin decoherence in a disordered field and further uncover the effects of the disorder.

In this work, we explore the dynamics of the XXZ CSM in a constant field, as well as a disordered field, and investigate how to improve the ability of central spin to resist decoherence. Although the Bethe ansatz method has given the exact solutions of CSM, its calculation process is very complicated. Therefore, we will use the state recurrence method to obtain the exact solution of the XXZ central spin model in the constant magnetic field. This method greatly simplifies the calculation process through simple closed-form expressions [42]. For the disordered field, the exact dynamics cannot be obtained; therefore, we will use the exact diagonalization technique to simulate the dynamics of the system. The fidelity and the entanglement entropy will be used to measure the dynamic behaviour of the system. The fidelity can quantify the change of quantum state over a period of time and reflect the decoherence of the central spin. In addition, as an important quantum correlation, the entanglement has attracted much attention [43,44,45]. The paper is organized as follows: In Section 2, we describe the model and briefly introduce some its properties. In Section 3, we analyze the influence of the anisotropic parameter and magnetic field on the dynamics of the CSM in a completely polarized bath. In Section 3.1, we use the state recurrence method to obtain the exact dynamics of the CSM in a constant magnetic field and use fidelity to analyze the decoherence process for a completely polarized bath. In Section 3.2, we numerically analyze the influence of a disordered magnetic field on fidelity and entanglement entropy between the central spin and the bath spins. In order to highlight the characteristics of dynamics in a disordered field, we compare the results for a constant field with a disordered field. Finally, a brief summary is given in Section 4.

## 2. Model

We consider a central spin model in a magnetic field [31], which describes a single electron trapped in a quantum dot built on a substrate containing nuclear spin. Denoting by $S_{0}$ the central spin−1/2 on the dot and by $S_{i}$ the *i*-th bath spin−1/2, the interaction between them has the form $\propto S_{0}S_{i}$. Here, we consider the XXZ interaction; the corresponding Hamiltonian reads

(2) $$H=J_{c}\sum_{i=1}^{K}\left(S_{0}^{x}S_{i}^{x}+S_{0}^{y}S_{i}^{y}+\lambda S_{0}^{z}S_{i}^{z}\right)+\sum_{i=1}^{K}h_{i}S_{i}^{z},$$

where $S_{i}^{x,y,z}=\sigma_{i}^{x,y,z}/2$ denotes the spin operator (ℏ=1), $J_{c}$ is the coupling strength, and λ is the anisotropy parameter. Furthermore, *K* is the number of bath spins, so the total number of spins is N=K+1. For convenience, we set $J_{c}=2$. For the constant field, we set the field strength as $h_{i}=2B\;\forall \;i$. For the disordered field, the random field strengths $h_{i}$ are uniformly distributed (i.e., $h_{i}\in \left[-W,W\right]$, where *W* represents the disorder strength).

As λ=1, the anisotropic central spin model degenerates into an isotropic model, which describes the Fermi contact hyperfine interaction between an electron spin and nuclear spins. In fact, in a real system, some additional effects will also influence the dynamics of the nuclear spin; therefore, over a longer time scale $\tau_{dd}$ ($\tau_{dd}\approx 10^{-4}$ s) in which typical GaAs dots are given directly by the inverse width of the nuclear magnetic resonance (NMR) line [46]), the dipole-dipole interaction will become dominant. The addition of an anisotropic parameter makes the model more rich in physical meaning. Changing the anisotropic parameter will cause the main interaction between electron spin and bath spins to change in three sources: (i) The Fermi contact hyperfine interaction, (ii) the dipole-dipole interaction, and (iii) the coupling of the orbital angular momentum to the nuclear spin [47]. In general, the Fermi contact hyperfine interaction provides the largest energy scale of the three contributions, but the contribution of the other interactions can be changed by the anisotropic parameter, such that the Hamiltonian corresponds to different models; for example, λ=1 is equivalent to electrons, λ=0.5 is a light hole, and λ=∞ is a heavy hole [23]. Note that the anisotropic parameter λ plays an important role in the quantum dot model; for this reason, we will investigate the influence of the anisotropic parameter in CSM.

## 3. Decoherence Dynamics

In this section, we consider an initial state as follows:

(3) $$|\psi_{0}\rangle =|⇓\rangle ⊗|\psi_{b}\rangle ,$$

where the bath spins are completely polarized $|\psi_{b}\rangle =|↑↑\cdots ↑\rangle$, and the central spin is spin-down |⇓〉. According to the Schrödinger equation in vector form,

(4) $$i\hbar \frac{\partial |\psi (t)\rangle }{\partial t}=H\psi \left(t\right).$$

When the Hamiltonian does not depend on time, the solution of the equation gives the quantum state at any time

(5) $$|\psi (t)\rangle =\exp(-iHt)|\psi_{0}\rangle ,$$

and the reduced density matrix of the central spin is

(6) $$\rho_{c}\left(t\right)={\mathrm{Tr}}_{b}\left(e^{-iHt}\rho_{0}e^{iHt}\right),$$

where $\rho_{0}=|\psi_{0}\rangle \langle \psi_{0}|$ and ${\mathrm{Tr}}_{b}$ denotes the partial trace over the bath degrees of freedom.

### 3.1. Constant Magnetic Field

For a constant magnetic field, we set $h_{i}=2B\;\forall \;i$, where *B* controls the magnetic field strength along the *z*-axis. By introducing a large spin operator $J=\sum_{j=1}^{K}S_{j}$ and setting $J_{c}=2$, Equation (2) is transformed as follows:

(7) $$H=S_{0}^{+}J^{-}+S_{0}^{-}J^{+}+2\lambda S_{0}^{z}J^{z}+2BJ^{z}.$$

The exact dynamics are obtained by using the state recurrence method, where the quantum state is given by (see Appendix A for details):

(8) $$|\psi (t)\rangle =P_{n}^{↓}|⇓\rangle |n\rangle +P_{n}^{↑}|⇑\rangle |n\rangle ,$$

and the coefficients are

(9) $$\begin{array}{cc} P_{n}^{↓} & =\cos\left(\frac{\Omega_{n-1}t}{2}\right)+\frac{\Delta_{n-1}}{\Omega_{n-1}}i\sin\left(\frac{\Omega_{n-1}t}{2}\right),\\ P_{n}^{↑} & =-\frac{2\sqrt{a_{n}}}{\Omega_{n-1}}i\sin\left(\frac{\Omega_{n-1}t}{2}\right), \end{array}$$

where

(10) $$\begin{array}{cc} \; & a_{n}=n\left(K-n+1\right),\\ \; & \omega_{n}^{\pm }=\left(2B\pm \lambda \right)\left(-\frac{K}{2}+n\right),\\ \; & \Delta_{n-1}=\omega_{n-1}^{+}-\omega_{n}^{-},\\ \; & \Omega_{n}=\sqrt{{\left(\omega_{n+1}^{-}+\omega_{n}^{+}\right)}^{2}+4\left(a_{n+1}-\omega_{n+1}^{-}\omega_{n}^{+}\right)}. \end{array}$$

For fully polarized bath spins, n=K; thus, $|\psi_{0}\rangle =|⇓\rangle ⊗|\underset{K}{\underset{︸}{↑↑\cdots ↑}}\rangle =|⇓\rangle ⊗|K\rangle$, and the wave function at any time is

(11) $$|\psi (t)\rangle =P_{K}^{↓}|⇓\rangle |K\rangle +P_{K}^{↑}|⇑\rangle |K\rangle .$$

The reduced density matrix of the central spin is

(12) $$\rho_{c}\left(t\right)={\mathrm{Tr}}_{b}|\psi (t)\rangle \langle \psi (t)|.$$

Fidelity, a measure of similarity between two quantum states, can be used to quantitatively describe the decoherence by calculating it between the initial central spin state and the state after evolution; that is,

(13) $$F\left(\rho_{1},\rho_{2}\right)={\left[\mathrm{Tr}{\left(\sqrt{\rho_{1}}\rho_{2}\sqrt{\rho_{1}}\right)}^{\frac{1}{2}}\right]}^{2},$$

where we set $\rho_{1}=\rho_{c}\left(0\right)$ and $\rho_{2}=\rho_{c}\left(t\right)$. Substituting Equation (12) into Equation (13), the fidelity reduces to

(14) $$F=1-\frac{2K}{\omega^{2}}+\frac{2K}{\omega^{2}}\cos\left(\omega t\right),$$

where $\omega =\sqrt{4K+{\left(2B-K\lambda +\lambda \right)}^{2}}$ determines the oscillation frequency and the amplitude. As the maximum fidelity is 1, we use the minimum fidelity to reflect the decoherence of the central spin:

(15) $$F_{\min}=1-\frac{4K}{\omega^{2}}=\frac{{\left(2B-K\lambda +\lambda \right)}^{2}}{4K+{\left(2B-K\lambda +\lambda \right)}^{2}}.$$

The value of $F_{\min}$ depends on several parameters, including the magnetic field strength *B*, the anisotropic parameter λ, and the number of bath spins *K*.

**Figure 1:**
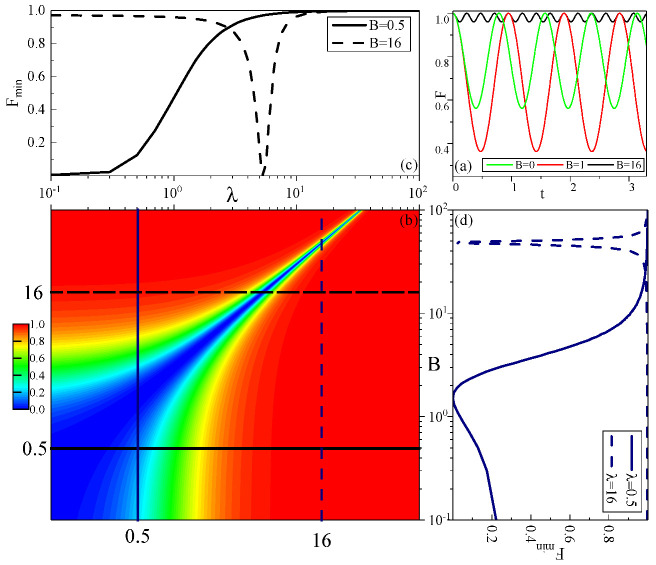
(Color online) Fidelity for the constant magnetic field (N=8): (**a**) The evolution of fidelity at different magnetic field strength *B* for λ=1. (**b**) The minimum value of fidelity $F_{\min}$ plotted versus the magnetic field strength *B* and anisotropy parameter λ. (**c**,**d**) are sections of (**b**) in the λ and *B* axes, respectively.

Figure 1 depicts the above results. In Figure 1a, the evolution of fidelity displays a periodic oscillation with no decay. Figure 1b shows the effect of magnetic field strength *B* and anisotropic parameter λ on the minimum fidelity $F_{\min}$, and sections of this are displayed in Figure 1c (where B=0.5 and 16.0) and Figure 1d (where λ=0.5 and 16.0). When the number of bath spins *K* is constant, the anisotropic parameter λ and magnetic field strength *B* determine the value of $F_{\min}$. When *B* and λ satisfy B=(Kλ−λ)/2, $F_{\min}$ achieves the minimum value of zero, and $F_{\min}$ changes dramatically near this minimum value. For a light hole (λ=0.5), a small magnetic field can have a big effect on $F_{\min}$, but we can infer that the magnetic field has little effect on $F_{\min}$ for a heavy hole λ→∞.

The oscillation frequency ω strongly depends on λ and *B*. Under the condition 2B−Kλ+λ=0, the frequency is minimized $\omega_{\min}=2\sqrt{K}$. Deviation of *B* or λ from this condition both causes the increase of frequency, where λ has a bigger effect on frequency for the number of bath spins K>3. In addition, when λ=1 and B=0, the model reduces to an isotropic model without a magnetic field; the oscillation frequency is simplified to ω=K+1=N and the minimum fidelity is $F_{\min}={\left(\frac{K-1}{K+1}\right)}^{2}={\left(1-\frac{2}{N}\right)}^{2}$, which is consistent with [18]. The frequency from $\omega_{\min}=2\sqrt{K}$ to ω=K+1 shows the transformation from the power law to the linear law and illustrates that the anisotropic parameter λ and the magnetic field *B* significantly affect the dynamic behaviour of the central spin.

### 3.2. Disordered Magnetic Field

Unlike the case of a constant field, the introduction of a disordered field complicates the dynamics of the system and the exact solutions cannot be obtained. Thus, we apply exact diagonalization techniques to calculate them. We randomly choose $h_{i}$ (i.e., uniformly distributed in [−W,W]) to simulate a disordered field. The dimension of the Hilbert space grows exponentially with an increase in the number of particles, but as $[H,S_{\mathrm{tot}}^{z}]=0$ (which means the *z* component of the total spin operator $S_{\mathrm{tot}}^{z}=S_{0}^{z}+\sum_{i=1}^{N}S_{i}^{z}$ is conserved), we can choose the conserved subspace of total spin to simplify the calculation. For the initial state $|\psi_{0}\rangle =|⇓\rangle ⊗{|↑↑\cdots ↑\rangle }_{K}$, the eigenvalue of the $S_{\mathrm{tot}}^{z}$ is $s_{\mathrm{tot}}^{z}=\left(K-1\right)/2$; thus, the eigenbasis of the subspace ν is $|\nu_{i}\rangle ={|↑\rangle }^{⊗(i-1)}|↓\rangle {|↑\rangle }^{⊗(N-i)}$, (i=1,2,3,...,N). In the subspace ν, the Hamiltonian can be rewritten as

(16) $$H_{\mathrm{sub}}=\nu^{†}H\nu .$$

In addition, the initial state is $|\psi_{0}\rangle =|\nu_{1}\rangle$; thus, the initial state can be rewritten as $|\psi_{0}^{\prime }\rangle ={\left(\begin{array}{cc} 1 & a \end{array}\right)}^{T}$ in the subspace, where *a* is a 1×K zero matrix. After a time interval *t*, the state becomes

(17) $$|\psi {\left(t\right)}^{\prime }\rangle =\exp\left(-iH_{\mathrm{sub}}t\right)|\psi_{0}^{\prime }\rangle =\sum_{n}e^{-iE_{n}t}|\phi_{n}\rangle \langle \phi_{n}|\psi_{0}^{\prime }\rangle ,$$

where $|\phi_{n}\rangle$ and $E_{n}$ are the eigenstates and eigenvalues of $H_{\mathrm{sub}}$. Converting to Hilbert space, we can obtain the full expression $|\psi (t)\rangle =\nu |\psi {\left(t\right)}^{\prime }\rangle$. As the initial bath is completely polarized, the dimension of the subspace is small, which greatly simplifies the calculation process.

#### 3.2.1. Fidelity

**Figure 2:**
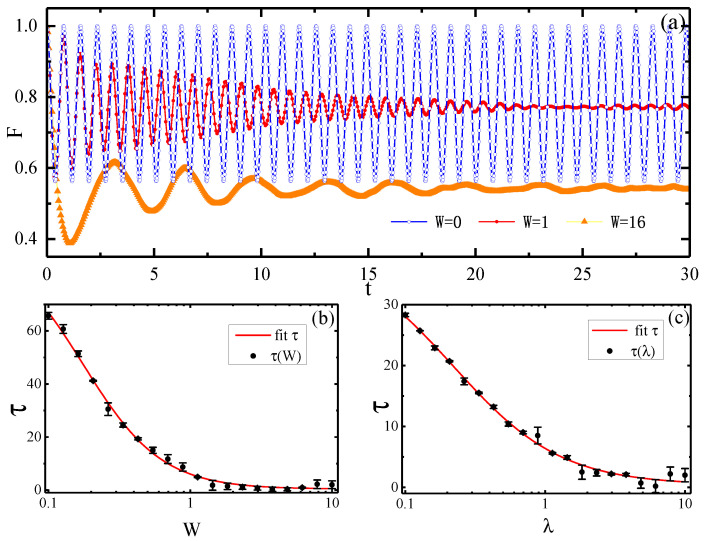
(Color online) (**a**) The evolution of fidelity for different disorder strengths *W* with λ=1. (**b**,**c**) show the decoherence time versus the disorder strength *W* and the anisotropy parameter λ, respectively. In (**b**), λ=1. In (**c**), W=1.

In Figure 2, we display the evolution of fidelity with different disorder strengths. When W=0, there is no interference from the external field and *F* oscillates within a certain range, which gives the results of Section 3.1. However, for W≠0, the addition of a disordered field causes the oscillation of fidelity to decay to a stable value. Furthermore, the relaxation time from the initial value of *F* to 1/e of this value is affected by the disorder strength *W* and the anisotropic parameter λ. In Figure 2b, we simulate the variation of the relaxation time with the disorder strength *W*. When the disorder is weak (W=0.1), the central spin relaxation time is very long. However, when the disorder strength is closer to 1.0, the relaxation time of the central spin is sharply shortened. This means a strong disorder can speed up the central spin relaxation process to reach a steady state. Similarly, in Figure 2c, keeping the disorder strength W=1, similar results are obtained by exploring the influence of the anisotropic parameter on relaxation time. A larger λ means a larger interaction intensity, which will also accelerate the relaxation process.

**Figure 3:**
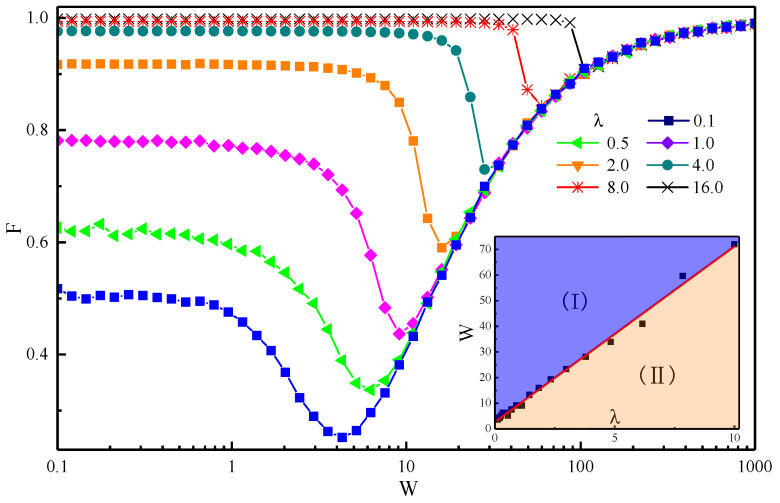
(Color online) Fidelity versus the disorder strength *W* for different values of the anisotropy parameter λ after a long time $t=10^{16}$ with the number of spins N=8. The bottom right inset shows the valley of different *F*–*W* curves correspond to *W* and λ, which is further divided into two regions, according to the valley value.

The value of *F* exactly reflects the decoherence of the central spin. The closer *F* is to 1, the smaller the difference between the present state and the initial state of the central spin is. The magnitude of the disorder strength and the anisotropic parameter will affect the value of fidelity after a long enough time; Figure 3 clearly shows this result. When the disorder strength is small, the anisotropic parameter has a great influence on *F*. At this time, the interaction between spins is dominant. However, an increase of *W* always causes the *F* to become equal for different values of λ. In other words, when the disorder strength is large enough, the fidelity of the final state will be independent of the anisotropic parameter λ and is only affected by the disorder strength *W*; therefore, disorder intensity plays a dominant role in the evolution of the system. Continuous enhancement of the disorder strength will improve the value of fidelity, thus weakening the degree of decoherence of the system in this area.

The bottom right inset of Figure 3 shows the movement of the valley value of the *F*–*W* curve through the disorder strength *W* and the anisotropic parameter λ. It can be seen that *W* is proportional to λ at the point of different valleys. That is, after the valley point, all of the curves overlap; but, with an increase of λ, the corresponding *W* of the valley point increases in direct proportion to λ. The movement of the valley indicates that the influence of *W* and λ on the degree of decoherence is competitive. When λ is small, a low disorder strength can be the main factor affecting the decoherence. When λ is large, the decoherence is affected by λ over a large range and the disorder strength only becomes the main factor affecting the decoherence if it is very large. The inset figure can be divided into two regions: The fidelity will be independent of the anisotropic parameter λ in area (I), but not in area (II).

Comparing Figure 3 with Figure 1, we find that the central spin in a constant magnetic field greatly differs from that in a disordered magnetic field. In the disordered field, the variation of *F* with *W* under different values of λ will converge to a curve after the valley, which means that the fidelity of the central spin will eventually show the same behaviour after the valley before the evolution of the system freezes (F=1). Compared with the constant field, the fidelity is independent of the anisotropic parameter λ only if the magnetic field is strong enough to freeze the evolution of the system ($F_{\min}=1$). Therefore, the range which is independent of anisotropy parameter in the disordered field is larger than that in the constant field. This means we can manipulate the quantum state only by adjusting the disorder strength *W*—without considering the form of interaction (this form is reflected by different λ)—to achieve the purpose of inhibiting central spin decoherence.

#### 3.2.2. Entanglement Entropy

**Figure 4:**
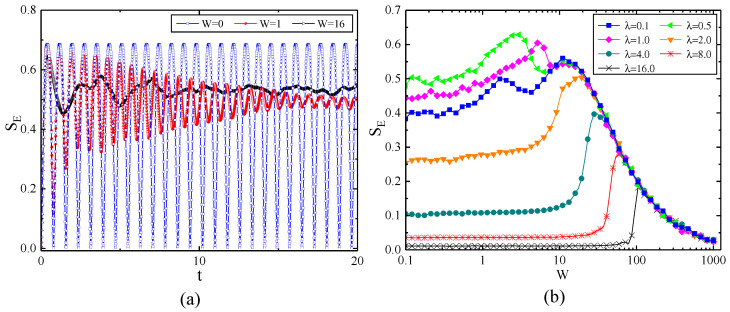
(Color online) (**a**) The evolution of entanglement entropy $S_{E}$ under different values of disorder strength *W* for λ=1. (**b**) The entanglement entropy versus the disorder strength *W* for different values of the anisotropy parameter λ for a long time $t=10^{16}$ and with the number of spins N=8.

When the central spin has decohered, the amount of decoherence is typically quantified by the entanglement entropy of its reduced density matrix. We consider the evolution of entanglement between the central spin and the bath spins. As we are considering an isolated quantum system, the entanglement entropy is given by the von Neumann entropy of the reduced density matrix. The reduced density matrix of the central spin is $\rho_{c}\left(t\right)={\mathrm{Tr}}_{b}|\psi (t)\rangle \langle \psi (t)|$; thus, the entanglement entropy is $S_{E}=-\mathrm{Tr}\left[\rho_{c}\log_{2}\rho_{c}\right]$. In the case of initially completely polarized bath spins, the change of entanglement entropy between the central spin and the bath spins with related parameters is shown in Figure 4.

The evolution of entanglement entropy is plotted in Figure 4a. The initial state of the system is a product state, but the system will become entangled through the interactions in the process of evolution. As W=0 corresponds to the case of no external disordered field, the evolution of entanglement entropy takes the form of periodic oscillation; however, the addition of a disordered external field will suppress this oscillation and eventually stabilize it at a fixed value. This is similar to the behaviour of fidelity. Figure 4b illustrates the entanglement entropy of the system after a long enough evolution, which means that the entangled entropy has grown to a stable saturation value. Consistent with fidelity, when *W* is small, the entanglement entropy is greatly affected by λ; however, when *W* becomes large to a certain extent, the entanglement entropy will be independent of the anisotropic parameter λ. By comparing Figure 3 with Figure 4b, the trends of $S_{E}$ and *F* are almost opposite, in other words, when the entanglement between the central spin and the bath spins is greater, the fidelity is smaller. The smaller fidelity means the degree of central spin decoherence becomes higher. The reason is that the hyperfine interaction makes the system entangled from product states and leads to the central spin decoherence. However, when λ is small, the trends of entanglement entropy and fidelity are not exactly opposite, which may imply a more complicated relationship between entanglement and decoherence.

## 4. Conclusions

By calculating the dynamic evolution of the central spin in a constant field and a disordered field under a completely polarized bath, we have investigated the properties of the central spin, which are summarized as follows. According to the exact solution of the XXZ central spin model in a constant field, the dynamic of the central spin is a simple, non-attenuating oscillation. The frequency and amplitude are affected by the anisotropic parameter λ and the magnetic field strength *B*. In addition, when λ=1 and B=0, the oscillation frequency is ω=K+1, which is linearly dependent on the number of bath spins *K*. However, in the special case 2B−Kλ+λ=0, the frequency is minimized $\omega_{\min}=2\sqrt{K}$, which indicates that the anisotropic parameter λ and the magnetic field *B* have strong power in regulating the oscillation frequency. In the disordered field, the fidelity passes a relaxation process (similar to a damping attenuation) and finally reaches a stable value. Furthermore, the relaxation time of this process is affected by the disorder strength and anisotropic parameter, where a larger anisotropic parameter or disorder strength can speed up the relaxation process. When the system reaches a steady state for a long enough time, the fidelity will become independent of the anisotropic parameter with an increase of the disorder strength, and enter a range where the disorder strength plays a dominant role in the evolution of the system. An increase of anisotropic parameter will increase the critical disorder intensity required to enter this range, which means there is a competitive relationship between the interaction and the disordered field. Therefore, we can change the fidelity by adjusting the disorder strength. A strong enough intensity of disorder can always enhance fidelity and suppress the decoherence of the central spin.

In addition, compared with the constant field, the disordered field can cause the fidelity to eventually maintain a stable value, rather than taking an oscillatory form, and the evolution of the system will be independent of the anisotropic parameter over a larger range. Therefore, in the process of dynamic evolution, the disordered field has good properties for retaining the initial state of the central spin. This not only eventually stabilizes the central spin, but also regulates the decoherence of central spin independently of anisotropic parameter over a larger range. In a real system, the types of interactions will change over time, such as the interaction from Fermi contact hyperfine interaction to the dipole-dipole interaction, which corresponds the change of the λ from isotropy to anisotropy. However, this change will not affect the dynamics of central spin when a strong enough disordered field is involved. This means that, by adding a disordered external field, we can eliminate the errors caused by changes in the internal parameter λ of the quantum system. If this property is used properly, it will have an important influence on regulating quantum states in the systems of quantum dots and NV centers.

## Appendix A

In this section, we present the calculation of the exact solution of XXZ CSM in a constant magnetic field. By introducing a large spin operator $J=\sum_{j=1}^{K}S_{j}$ and setting $J_{c}=2$, Equation (2) can be rewritten as

(A1) $$H=S_{0}^{+}J^{-}+S_{0}^{-}J^{+}+2\lambda S_{0}^{z}J^{z}+2BJ^{z}.$$

As the bath spins are prepared in a fully polarized state, we obtain the initial state

(A2) $$|\psi_{0}\rangle =|⇓\rangle ⊗|\psi_{b}\rangle =|⇓\rangle ⊗|n\rangle ,$$

where $|n\rangle =|\frac{K}{2},n-\frac{K}{2}\rangle$ is the eigenstate of $J^{2}$ and $J^{z}$. After a time interval *t*, we have

(A3) $$|\psi (t)\rangle =e^{-iHt}|\psi_{0}\rangle =\sum_{m=0}^{\infty }\frac{{\left(-it\right)}^{m}}{m!}H^{m}|\psi_{0}\rangle =\sum_{m=0}^{\infty }\frac{{\left(-it\right)}^{m}}{m!}H^{m}|⇓\rangle |n\rangle .$$

According to the following eigenstate relation of angular momentum operators

(A4) $$\begin{array}{cc} \; & J^{-}|n\rangle =\sqrt{a_{n}}|n-1\rangle ,\\ \; & J^{+}|n\rangle =\sqrt{a_{n+1}}|n+1\rangle ,\\ \; & J^{z}|n\rangle =\left(-K/2+n\right)|n\rangle , \end{array}$$

where $a_{n}=n\left(K-n+1\right)$, applying *H* on |⇑〉|n〉 and |⇓〉|n〉, we obtain

(A5) $$\begin{array}{cc} H|⇑\rangle |n\rangle & =\omega_{n}^{+}|⇑\rangle |n\rangle +\sqrt{a_{n+1}}|⇓\rangle |n+1\rangle ,\\ H|⇓\rangle |n\rangle & =\omega_{n}^{-}|⇓\rangle |n\rangle +\sqrt{a_{n}}|⇑\rangle |n-1\rangle , \end{array}$$

we denote $\omega_{n}^{\pm }=\left(2B\pm \lambda \right)\left(-\frac{K}{2}+n\right)$. Again applying *H*,

(A6) $$\begin{array}{cc} H^{2} & |⇑\rangle |n\rangle =\left(\omega_{n}^{+}+\omega_{n+1}^{-}\right)H|⇑\rangle |n\rangle +\left(a_{n+1}-\omega_{n+1}^{-}\omega_{n}^{+}\right)|⇑\rangle |n\rangle ,\\ H^{2} & |⇓\rangle |n\rangle =\left(\omega_{n}^{-}+\omega_{n-1}^{+}\right)H|⇓\rangle |n\rangle +\left(a_{n}-\omega_{n-1}^{+}\omega_{n}^{-}\right)|⇓\rangle |n\rangle . \end{array}$$

We obtain the following recurrence relations by applying the Hamiltonian on the |⇓〉|n〉 multiple times:

(A7) $$t_{m+2}-\left(\omega_{n}^{-}+\omega_{n-1}^{+}\right)t_{m+1}-\left(a_{n}-\omega_{n}^{-}\omega_{n-1}^{+}\right)t_{m}=0,$$

where $t_{m}=H^{m}|⇓\rangle |n\rangle$ and $t_{0}=|⇓\rangle |n\rangle ,t_{1}=\omega_{n}^{-}|⇓\rangle |n\rangle +\sqrt{a_{n}}|⇑\rangle |n-1\rangle .$ The characteristic equation of the recurrence relation $t_{m}$ is

(A8) $$\xi^{2}-\left(\omega_{n}^{-}+\omega_{n-1}^{+}\right)\xi -\left(a_{n}-\omega_{n}^{-}\omega_{n-1}^{+}\right)=0.$$

The characteristic root of Equation (A8) is

(A9) $$\xi_{1,2}=\frac{\left(\omega_{n}^{-}+\omega_{n-1}^{+}\right)\pm \Omega_{n-1}}{2},$$

where

(A10) $$\Omega_{n}=\sqrt{{\left(\omega_{n+1}^{-}+\omega_{n}^{+}\right)}^{2}+4\left(a_{n+1}-\omega_{n+1}^{-}\omega_{n}^{+}\right)}.$$

Therefore, $t_{m}$ is given by

(A11) $$t_{m}=H^{m}|⇓\rangle |n\rangle =\frac{t_{1}-\xi_{2}t_{0}}{\Omega_{n-1}}\xi_{1}^{m}+\frac{-t_{1}+\xi_{1}t_{0}}{\Omega_{n-1}}\xi_{2}^{m}.$$

By substituting the expression of $t_{m}$ into Equation (A3), we obtain

(A12) $$\begin{array}{cc} |\psi (t)\rangle & =\sum_{m=0}^{\infty }\frac{{\left(-it\right)}^{m}}{m!}H^{m}|⇓\rangle |n\rangle \\ & =\sum_{m=0}^{\infty }\frac{{\left(-it\right)}^{m}}{m!}\left(\frac{t_{1}-\xi_{2}t_{0}}{\Omega_{n-1}}\xi_{1}^{m}+\frac{-t_{1}+\xi_{1}t_{0}}{\Omega_{n-1}}\xi_{2}^{m}\right)\\ \; & =\frac{t_{1}-\xi_{2}t_{0}}{\Omega_{n-1}}\exp\left(-i\xi_{1}t\right)+\frac{-t_{1}+\xi_{1}t_{0}}{\Omega_{n-1}}\exp\left(-i\xi_{2}t\right). \end{array}$$

Substituting the initial condition $t_{0},t_{1}$ and $\xi_{1},\xi_{2}$ into the above formula, we get

(A13) $$|\psi (t)\rangle =P_{n}^{↓}|⇓\rangle |n\rangle +P_{n}^{↑}|⇑\rangle |n\rangle ,$$

where the parameters were denoted by

(A14) $$\begin{array}{cc} P_{n}^{↓} & =\cos\left(\frac{\Omega_{n-1}t}{2}\right)+\frac{\Delta_{n-1}}{\Omega_{n-1}}i\sin\left(\frac{\Omega_{n-1}t}{2}\right),\\ P_{n}^{↑} & =-\frac{2\sqrt{a_{n}}}{\Omega_{n-1}}i\sin\left(\frac{\Omega_{n-1}t}{2}\right), \end{array}$$

with

(A15) $$\begin{array}{cc} \; & a_{n}=n\left(K-n+1\right),\\ \; & \omega_{n}^{\pm }=\left(2B\pm \lambda \right)\left(-\frac{K}{2}+n\right),\\ \; & \Delta_{n}=\omega_{n}^{+}-\omega_{n+1}^{-},\\ \; & \Omega_{n}=\sqrt{{\left(\omega_{n+1}^{-}+\omega_{n}^{+}\right)}^{2}+4\left(a_{n+1}-\omega_{n+1}^{-}\omega_{n}^{+}\right)}. \end{array}$$

The reduced density matrix of the central spin is

(A16) $$\rho_{c}\left(t\right)=\left(\begin{array}{cc} a_{11} & a_{12}\\ a_{21} & a_{22} \end{array}\right).$$

For the initial state of the bath spins in the completely polarized bath n=K, we get the four matrix elements

(A17) $$\begin{array}{cc} \; & a_{11}\left(t\right)={\left|P_{n}^{↑}\right|}^{2}={\left|P_{K}^{↑}\right|}^{2}=\frac{4a_{K}}{\Omega_{K-1}^{2}}\sin^{2}\left(\frac{\Omega_{K-1}t}{2}\right),\\ \; & a_{22}\left(t\right)={\left|P_{n}^{↓}\right|}^{2}={\left|P_{K}^{↓}\right|}^{2}=\cos^{2}\left(\frac{\Omega_{K-1}t}{2}\right)+\frac{\Delta_{K-1}^{2}}{\Omega_{K-1}^{2}}\sin^{2}\left(\frac{\Omega_{K-1}t}{2}\right),\\ \; & a_{12}\left(t\right)=a_{21}\left(t\right)=0. \end{array}$$

The fidelity of central spin between $\rho_{c}\left(0\right)$ with $\rho_{c}\left(t\right)$ is

(A18) $$F\left(\rho_{c}\left(0\right),\rho_{c}\left(t\right)\right)={\left[\mathrm{Tr}{\left(\sqrt{\rho_{c}\left(0\right)}\rho_{c}\left(t\right)\sqrt{\rho_{c}\left(t\right)}\right)}^{\frac{1}{2}}\right]}^{2}=a_{22}\left(t\right)=1-\left(1-\frac{\Delta_{K-1}^{2}}{\Omega_{K-1}^{2}}\right)\sin^{2}\left(\frac{\Omega_{K-1}t}{2}\right).$$

Further simplification leads to

(A19) $$F=1-\frac{2K}{\omega^{2}}+\frac{2K}{\omega^{2}}\cos\left(\omega t\right),$$

where $\omega =\sqrt{4K+{\left(2B-K\lambda +\lambda \right)}^{2}}$.
